# Effective prognostic risk model with cuproptosis-related genes in laryngeal cancer

**DOI:** 10.1016/j.bjorl.2023.101384

**Published:** 2023-12-26

**Authors:** Cong Li, Yongzhi Zhu, Song Shi

**Affiliations:** a Shanghai Jiao Tong University School of Medicine, Tongren Hospital, Department of Otorhinolaryngology, Shanghai, China; b Zhengzhou Shuqing Medical College, Zhengzhou, China

**Keywords:** Cuproptosis, Risk model, Prognosis, Laryngeal cancer, Immune

## Abstract

**Objective:**

Laryngeal cancer, characterized by high recurrence rates and a lack of effective biomarkers, has been associated with cuproptosis, a regulated cell death process linked to cancer progression. In this study, we aimed to explore the roles of cuproptosis-related genes in laryngeal cancer and their potential as prognostic markers and therapeutic targets.

**Methods:**

We collected comprehensive data from The Cancer Genome Atlas and Gene Expression Omnibus databases, including gene expression profiles and clinical data of laryngeal cancer patients. Using clustering and gene analysis, we identified cuproptosis-related genes with prognostic significance. A risk model was constructed based on these genes, categorizing patients into high- and low-risk groups for outcome comparison. Univariate and multivariate analyses were conducted to identify independent prognostic factors, which were then incorporated into a nomogram. Gene Set Enrichment Analysis was employed to explore pathways distinguishing high- and low-risk groups.

**Results:**

Our risk model, based on four genes, including transmembrane 2, dishevelled binding antagonist of β-catenin 1, stathmin 2, and G protein-coupled receptor 173, revealed significant differences in patient outcomes between high- and low-risk groups. Independent prognostic factors were identified and integrated into a nomogram, providing a valuable tool for prognostic prediction. Gene Set Enrichment Analysis uncovered up-regulated pathways specifically associated with high-risk patient samples.

**Conclusion:**

This study highlights the potential of cuproptosis-related genes as valuable prognostic markers and promising therapeutic targets in the context of laryngeal cancer. This research sheds light on new avenues for understanding and managing this challenging disease.

**Level of evidence:**

Level 4.

## Highlights

• Cuproptosis genes stratified laryngeal cancer into diverse prognostic subtypes.
• TMEM2, DACT1, STMN2, and GPR173 form our prognostic model.
• TMEM2, DACT1, STMN2, GPR173 shape the risk model.
• High-risk samples upregulated angiogenesis, EMT, and KRAS pathways.
• Clinical_M, Clinical_cN, risk groups predict laryngeal cancer prognosis.

## Introduction

Laryngeal cancer is the second most common malignant tumor of the head and neck.^1^ The cure rate for early stage laryngeal cancer is higher than 80%, although the recurrence rate for advanced laryngeal cancer is 25%–50%.^2^, ^3^ Furthermore, the 5-year survival rate for stage IV laryngeal cancer patients is even lower at 40%,^2^ thus, an early diagnosis would increase the likelihood of curing the disease. Patients with an early diagnosis of laryngeal cancer can often be treated with surgery or radiation, which ultimately preserves the larynx. In contrast, patients with advanced disease often require multimodal treatment and are less often laryngeal preserving.^4^ Currently, there are no biomarkers for the early and prognostic detection of laryngeal cancer. Therefore, here we investigate whether clinical markers widely used in a variety of diseases could also play a role in laryngeal cancer.

Cell death caused by abnormal iron levels is called ferroptosis,^5^ and recent studies have now also identified intracellular copper-induced cell death called cuproptosis.^6^ Copper can bind to the lipid-acylated components of the tricarboxylic acid cycle, causing proteotoxic stress and eventually inducing ferroptosis in cells distinct from other apoptotic pathways.^7^ Genes related to Cu^2+^ transport, such as recombinant copper transporter 1 (SLC31A1), affect dietary copper uptake and have a possible role in regulating cuproptosis.^8^ Additional studies have demonstrated the potential impact of cuproptosis-related genes on the immune microenvironment, clinicopathological features, and prognosis of triple-negative breast cancer.^9^ For example, Huang et al. developed a scoring system to predict prognosis and immunity in patients with squamous cell carcinoma of the head and neck based on cuproptosis-related genes, which showed good predictive results.^10^ However, no risk models have yet been constructed using copper death-related genes that can predict the prognoses of patients with laryngeal cancer.

In the present study, we investigated expression patterns of cuproptosis-related genes in patients with laryngeal cancer and normal participants. We expect the risk model constructed using cuproptosis-related genes to have good prognosis predictive performance for laryngeal cancer patients and provide new therapeutic methods for laryngeal cancer therapy.

## Methods

### Data collection and preprocessing

Head and neck squamous cell carcinoma gene expression and clinical follow-up data from The Cancer Genome Atlas (TCGA) database were downloaded on July 20, 2022. Gene expression data were normalized to log_2_(FPKM + 1). Furthermore, we screened samples based on the following criteria: 1) The site of Head and Neck Squamous Cell Carcinoma was the larynx, 2) Patients had well-preserved prognostic information, and 3) Patients had a non-zero survival time. As a result, a total of 81 primary laryngeal cancer samples with prognostic information were obtained.

The laryngeal cancer dataset GSE27020^11^ from the NCBI Gene Expression Omnibus (GEO) database was downloaded, and GSE27020, which included 109 laryngeal cancer samples with prognostic information, was used for validation analysis. Furthermore, 19 cuproptosis-related genes were collected from published literature.^6^

### Identification of cuproptosis subtypes in laryngeal cancer

The expression levels of cuproptosis-related genes were compared between laryngeal cancer patients and healthy participants, whilst Pearson correlations between cuproptosis-related genes were also calculated. Subsequently, based on the differential expression of cuproptosis-related genes between laryngeal cancer patients and healthy participants, we performed tumor subtype analysis of the samples using unsupervised hierarchical clustering R3.6.1 ConsensusClusterPlus (version 1.54.0).^12^ Finally, the most suitable cuproptosis subtype was obtained when the K-value was set between 2 and 6.

### Prognostic analysis of of cuproptosis subtypes

In the R package (version 1.36.2), the Gene Set Variation Analysis (GSVA) algorithm^13^ was used to calculate enrichment scores between samples from different subtype groups and to then represent the cuproptosis score for each sample. The enrichment scores between different subtypes were investigated using the Wilcoxon test to justify the cuproptosis subtypes.

Subsequently, the Kaplan-Meier curve^14^ was used to assess the contrast in prognosis between the subtype samples. Furthermore, we compared differences in the clinical information of patients with different subtypes.

### Comparison of immune microenvironment between cuproptosis subtypes

CIBERSORT^15^ was used to calculate the proportions of 22 immune cell compositions in both subtype groups. The Wilcoxon test was used to compare differences in infiltration between the groups and subsequently, the stromal and immune scores of the tumor samples were determined using the ESTIMATE algorithm.^16^

### Identification specific and prognostic genes between cuproptosis subtypes

To observe the possible different molecular mechanisms between various cuproptosis subtypes, we used linear regression and classical Bayesian methods in the limma package (version 3.10.3)^17^ to perform differential gene expression analysis for each subtype. Subsequently, Benjamini and Hochberg were used to correct the results, and the adj.P.Value was obtained. Next, Differentially Expressed Genes (DEGs) with an adj.P.Value < 0.05 and |log2FC| > 0.5 were defined as inter-subtype specific expressed genes.

Furthermore, we screened genes significantly related to prognosis from specific expressed genes using univariate Cox regression analysis of the survival package in R3.6.1 (version 2.41-1).^14^ The significance threshold was set at *p* < 0.05.

### Construction of prognostic model

A risk model was constructed with stepwise Cox regression analysis of the Survminer package in R3.6.1 (version 0.4.9) based on genes significantly associated with prognosis. The risk score was calculated using the following formula: Risk score = h_0_(t) × exp ( β_1_X_1_ + β_2_X_2_ + … +β_n_X_n_ ).

In this formula, β, h_0_(t), and h(t,X) refer to the regression coefficients, baseline risk function, and risk function associated with X (covariate), respectively, at time, t.

For the samples’ risk scores in TCGA and GEO datasets, samples were divided into high- (>median risk scores) and low-risk groups (≤median risk score). Prognostic variability between different risk groups was assessed using the Kaplan-Meier curve method.^14^

### Construction of nomogram model

Clinical information was compared among the different risk groups. Independent prognostic factors were then screened using univariate and multivariate Cox regression using *p* < 0.05 as a threshold, and forest plots were drawn. We then collated independent prognostic factors and constructed a nomogram using the rms package (version 5.1-2).^18^

### Comparison of immune checkpoint genes and human leukocyte antigen (HLA) family genes between patients in different risk groups

Based on the Wilcoxon test, the expression of immune checkpoint genes and HLA family genes was compared between patients in different risk groups.

### Gene set enrichment analysis (GSEA)

Enrichment of significant hallmark gene sets (h.all.v7.4. symbols) between different risk groups were investigated using GSEA. The threshold for screening significantly different genes set was set at *p* < 0.05 and ǀNESǀ > 1.

### Association of risk group with cuproptosis subtypes

Using the ggalluvial package (version 0.12.3) in R3.6.1, we compared the proportions of the two cuproptosis subtypes between the different risk groups and investigated the association between these subtypes and subgroups.

## Results

### Expression pattern of cuproptosis-related genes in laryngeal cancer patients

**Figure 1:**
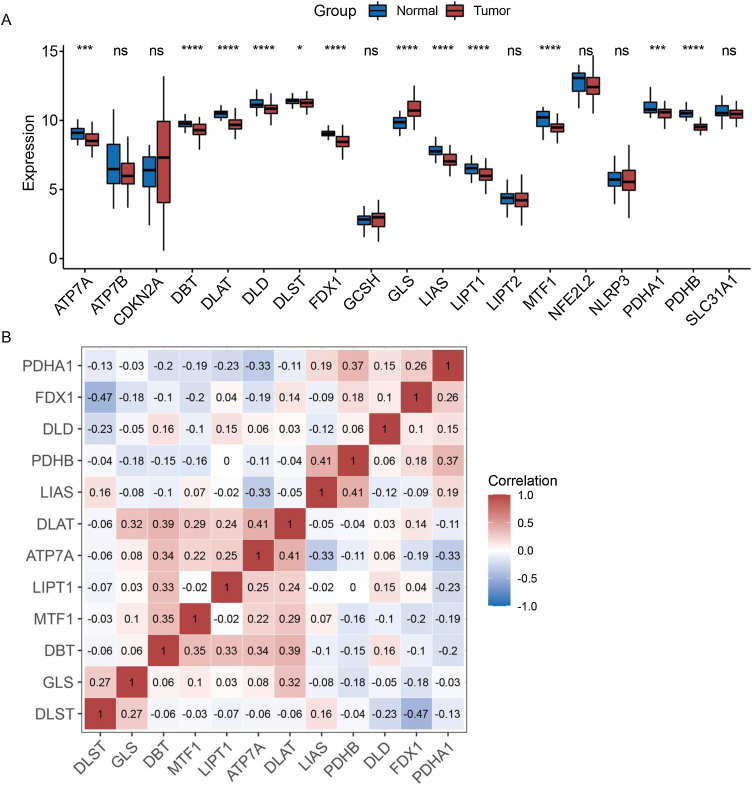
Expression patterns of cuproptosis-related genes. (A) Expression of cuproptosis-related genes in laryngeal cancer and normal samples. (B) Correlation analysis of differentially expressed cuproptosis-related genes.

The expression patterns of 19 cuproptosis-related genes were analyzed across laryngeal cancer patients and healthy participants, with 12 of these genes showing significantly different expression patterns (Fig. 1A) (*p* < 0.05). Furthermore, we investigated the correlations between these 12 cuproptosis-related genes and found negative correlations between the majority of them (Fig. 1B).

### Two cuproptosis subtypes were identified in laryngeal cancer samples

**Figure 2:**
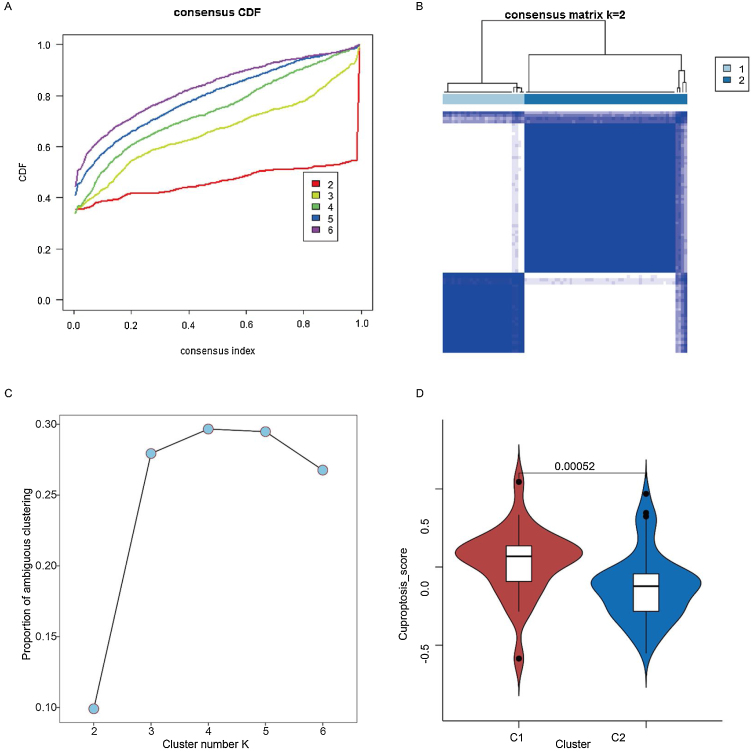
Identification of two cuproptosis subtypes in laryngeal cancer samples. (A) Cumulative Distribution Function (CDF) for consensus clustering. (B) Consensus clustering matrix when K is 2. (C) Proportion of ambiguous clustering analysis. (D) Cuproptosis scores of C1 and C2 cuproptosis subtypes.

Based on the 12 cuproptosis-related genes differentially expressed in healthy and cancerous samples, unsupervised clustering analysis of tumor samples was performed. Subsequently, two of these subtypes were chosen as the best K-values (Fig. 2 A–C). In addition, to further validate the plausibility of the subtypes, we compared cuproptosis scores between subtypes, with these results restricting the cuproptosis scores of patients with the C1 subtype (27 samples) to be significantly higher than the C2 subtype (54 samples) (Fig. 2D) (*p* < 0.05).

### Correlation of cuproptosis subtypes with clinical features

**Figure 3:**
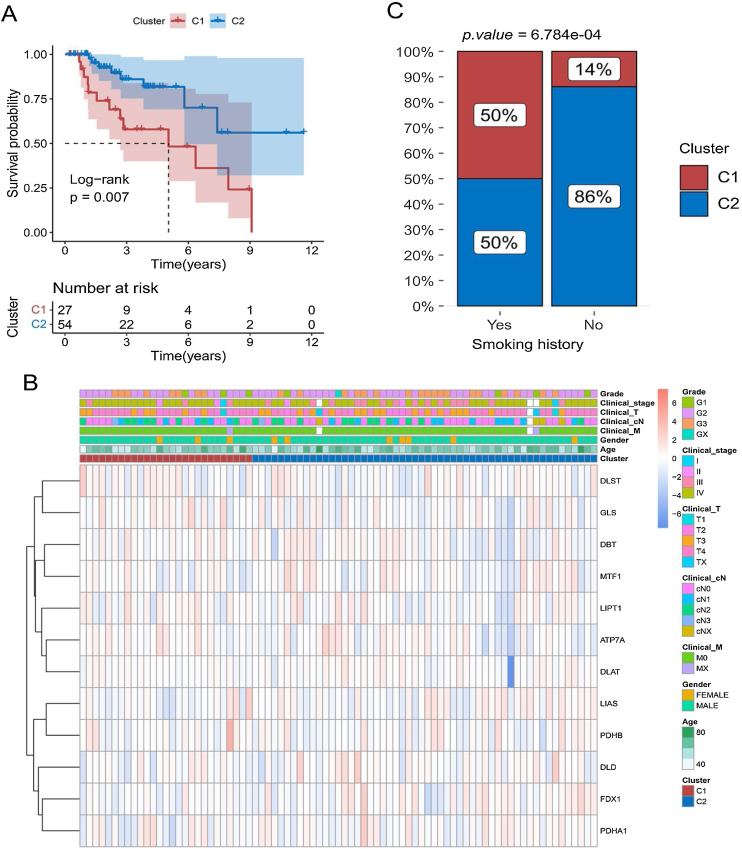
Relationship between cuproptosis subtypes and survival of laryngeal cancer patients. (A) Comparison of survival differences between patients with C1 and C2 subtypes using Kaplan-Meier curve. (B) Heat map of the distribution of expression levels of 12 differential cuproptosis-related genes in subtype samples. (C) Comparison of the proportion of patients with C1 and C2 subtypes of laryngeal cancer among patients with different smoking histories.

Kaplan-Meier curves showed that patients with laryngeal cancer in the C2 subtype had a significantly better prognosis than those in the C1 subtype (Fig. 3A) (*p* < 0.05). We also compared the expression patterns of 12 copper death genes in patients of both subtypes (Fig. 3B). Subsequently, the proportions of patients with the C1 and C2 subtypes were also compared under different clinical characteristics. The proportion of C2 subtypes was higher for patients with a history of smoking (Fig. 3C and Supplementary Fig. 1).

### Immune analysis

**Figure 4:**
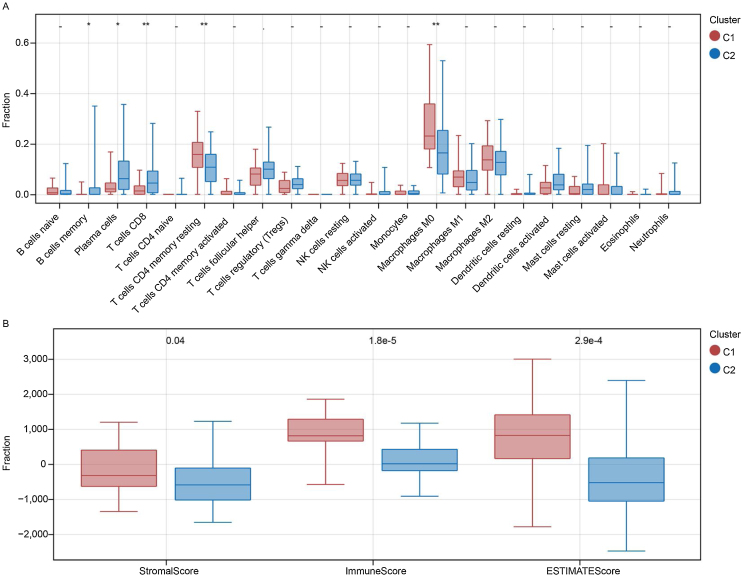
Immune analysis. (A) Fraction of 22 immune cells in C1 and C2 samples. (B) Comparison of the stromal, immune, and ESTIMATE scores between patients in the C1 and C2 subtypes.

The fraction of immune cells in patients with subtypes C1 and C2 was compared. Between the two subtypes, 5 of the 22 immune cells were significantly different (Fig. 4A). Specifically, the proportion of plasma cells, memory B cells, and CD8 T cells was higher in the C2 subtype, whereas the proportion of M0 macrophages and CD4 memory resting T-cells was higher in the C1 subtype (*p* < 0.05). Further investigation also indicated that stromal, immune, and ESTIMATE scores were always higher in the C1 subgroup than in the C2 subgroup (Fig. 4B).

### Screening for genes significantly associated with prognosis

**Figure 5:**
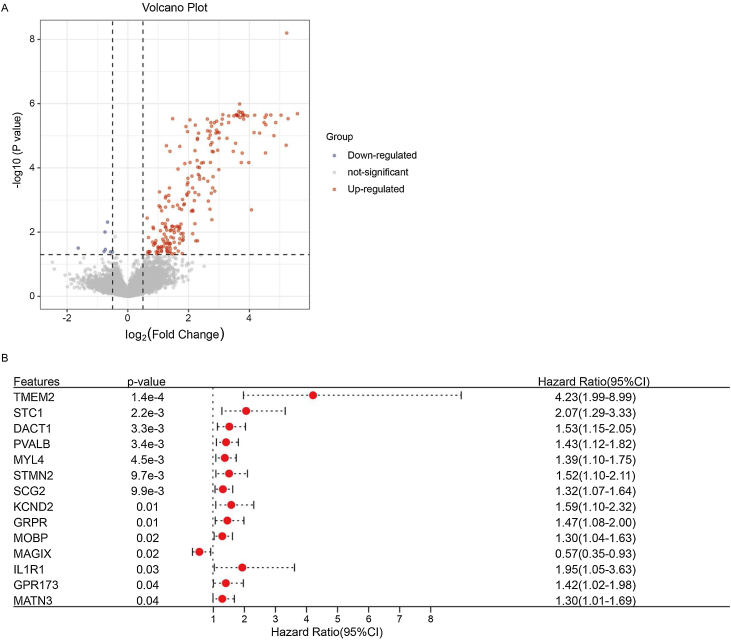
Identification of the prognostic genes of patients with laryngeal cancer. (A) Volcano map was used to show differentially expressed genes between C1 and C2 subtypes. (B) Forest plot was used to show prognosis-related genes screened by univariate Cox regression analysis.

By comparing the gene expression between subtypes C1 and C2, we obtained 218 DEGs (211 upregulated and 7 downregulated genes) (Fig. 5A, Supplementary Table 1). We screened the prognostic genes from 218 DEGs using a univariate Cox analysis (Fig. 5B). Finally, 14 genes were defined as significantly related to patient prognosis, of which 13 genes: Transmembrane 2 (TMEM2), Stanniocalcin 1 (STC1), Dishevelled binding Antagonist of β-Catenin 1 (DACT1), Parvalbumin (PVALB), Myosin Light chain 4 (MYL4), Stathmin 2 (STMN2), Secretogranin II (SCG2), Potassium voltage-gated Channel subfamily D member 2 (KCND2), Gastrin Releasing Peptide Receptor (GRPR), Myelin-associated Oligodendrocyte Basic Protein (MOBP), Interleukin 1 Receptor I (IL1R1), G Protein-coupled Receptor 173 (GPR173), and Matrilin-3 (MATN3); were risk factors, whilst 1 gene (MAGIX) was a protective factor.

### Constructing a risk model for patients with laryngeal cancer

**Figure 6:**
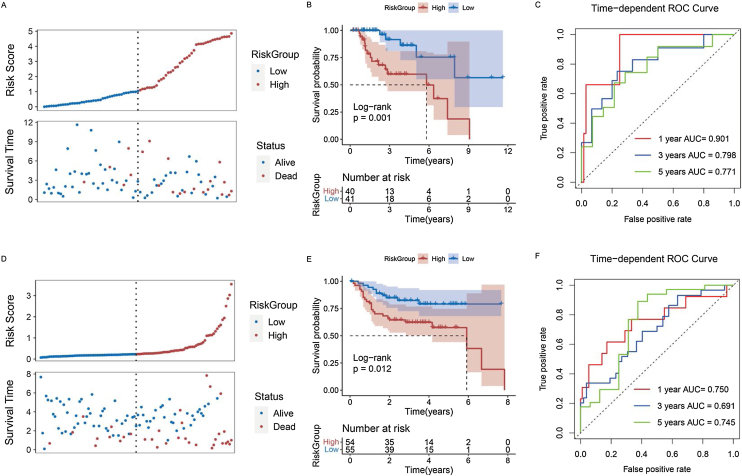
Predictive performance of risk model for prognosis of laryngeal cancer patients. (A) Survival status of patients with laryngeal cancer in high- and low-risk groups in TCGA database. (B) Kaplan-Meier curve of laryngeal cancer patients in high- and low-risk groups in TCGA database. (C) ROC curves were used to present the predictive performance of the risk model for 1-, 3-, and 5-year survival of patients with laryngeal cancer in TCGA database. (D) Survival status of patients with laryngeal cancer in high- and low-risk groups in GEO database. (E) Kaplan-Meier curve of laryngeal cancer patients in high- and low-risk groups in GEO database. (F) ROC curves were used to present the predictive performance of the risk model for 1-, 3-, and 5-year survival of patients with laryngeal cancer in GEO database.

We used the stepwise Cox regression algorithm to screen for the best combination of the 14 genes significantly associated with prognosis for risk model construction. TMEM2, DACT1, STMN2, and GPR173 were used to construct the risk model (Supplementary Fig. 2). After classifying the samples into different groups, we found that there were more deaths among the patients in the high-risk group (Fig. 6A) and moreover, the high-risk group had a worse prognosis (Fig. 6B). The Area Under the Curve (AUC) of the model predicting patients at 1, 3, and 5 years were 0.901, 0.798, and 0.771, respectively (Fig. 6C). Based on the median risk score, patients in the GEO dataset were grouped to validate the analysis results in the TCGA dataset. GEO results also indicated that the high-risk group had more deaths and a worse overall prognosis (Fig. 6 D,E). The Receiver Operating Characteristic (ROC) curve in the GEO dataset also proved to be a good prediction performance model (Fig. 6F).

### Nomogram was constructed with independent prognostic factors

**Figure 7:**
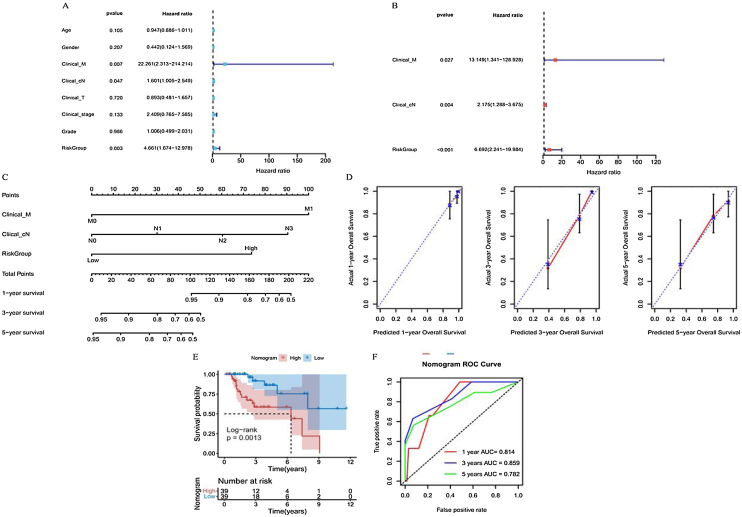
Predictive performance of nomogram model for prognosis of laryngeal cancer patients. (A and B) Univariate and multivariate Cox regression analysis to screen prognostic independent factors in patients with laryngeal cancer. (C) A nomogram constructed by the prognostic independent factors. (D) Calibration curves were used to demonstrate the difference between the 1-, 3-, and 5-year survival predicted by nomogram for patients with laryngeal cancer and the actual survival. (E) Kaplan-Meier curve of laryngeal cancer patients in high- and low-nomogram groups. (F) ROC curves were used to present the predictive performance of the nomogram model for 1-, 3-, and 5-year survival of patients with laryngeal cancer.

Primary Tumor (T), clinical regional lymph Nodes (cN), distant Metastasis (M), age, sex, clinical stage, grade, and risk groups were all included in the univariate Cox regression analysis. Significant results were further included in this analysis (Fig. 7A). Finally, distant metastasis M, clinical regional lymph nodes cN, and risk groups were identified as independent prognostic factors for patients with laryngeal cancer and were used to construct a subsequent nomogram (Fig. 7B). The C-index of this nomogram was 0.809 (Fig. 7C), and the calibration curve showed a good agreement between the predicted and observed values of the column line graph model for the prediction of overall survival at 1, 3, and 5 years in patients with laryngeal cancer (Fig. 7D).

Patients were then grouped based on this nomogram. The Kaplan-Meier curve indicated that the prognoses of laryngeal cancer patients in the high nomogram group were significantly poorer than those in the low nomogram group (Fig. 7E). In addition, the ROC curve further demonstrated that the prognosis of laryngeal cancer patients at 1, 3, and 5 years was well predicted using the nomogram, with respective AUCs of 0.814, 0.859, and 0.782 (Fig. 7F).

### Differences in immune checkpoint genes and HLA family genes between patients in different groups

The expression of Signal regulatory protein alpha (MYD1) and Receptor induced by lymphocyte activation (4-1BB) was significantly greater in the high-risk group than in the low-risk group (Supplementary Fig. 3A) (*p* < 0.05), although further studies showed no significant differences in HLA gene family expression between groups (Supplementary Fig. 3A) (*p* < 0.05).

### GSEA for high- and low-risk groups

**Figure 8:**
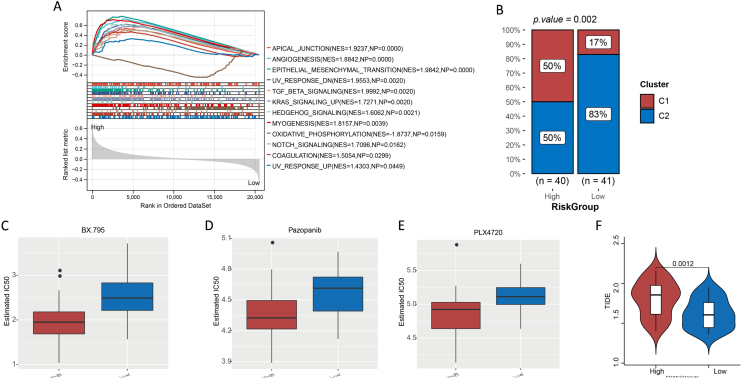
(A) Gene set enrichment analysis. (B) Proportion of cuproptosis subtypes C1 and C2 in patients in high- and low-risk groups. (C—F) Evaluation of treatment response in patients with laryngeal cancer. (C, D, and E) IC50 of BX.759, pazopanib, and PLX4720 for patients with laryngeal cancer in high- and low-risk groups. (F) TIDE score of patients with laryngeal cancer in high- and low-risk groups.

Hallmark gene sets were screened with *p* < 0.05 and ǀNESǀ > 1. Finally, we obtained 11 hallmark gene sets that were significantly enriched in the high-risk group, such as angiogenesis (NES = 1.8842, NP = 0.0000), epithelial-mesenchymal transition (NES = 1.9842, NP = 0.0000), TGF beta signaling (NES = 1.9992, NP = 0.0020), and Kirsten Rat Sarcoma viral oncogene homolog (KRAS) signaling (NES = 1.7271, NP = 0.0020) (Fig. 8A). Oxidative phosphorylation (NES = −1.8737, NP = 0.0159) was the only hallmark gene set that was significantly enriched in the low-risk group (Fig. 8A).

### Cuproptosis subtypes were associated with risk groups

For the different risk groups, we used the chi-square test to compare the proportions of subtypes C1 and C2. The results indicated that the proportion of patients with the C2 subtype was significantly higher for the low-risk group than in the high-risk group (Fig. 8B, *p* < 0.05).

### Differences in treatment sensitivity

We investigated the sensitivity of patients in the different risk groups to 138 chemotherapeutic agents. The IC50 results revealed a significant difference in the sensitivity of patients in the different groups to 37 chemotherapeutic agents (Supplementary Table 2). The top three most significant drugs were N-[3-[[5-Iodo-4-[[3-[(2-Thienylcarbonyl)amino]propyl]amino]-2-pyrimidinyl]amino]phenyl]-1-pyrrolidinecarboxamide (BX.795), pazopanib, and N-[3-[(5-Chloro-1H-pyrrolo[2,3-b]pyridin-3-yl)carbonyl]-2,4-difluorophenyl]-1-propanesulfonamide (PLX4720) (Fig. 8D–F). The Tumor Immune Dysfunction and Exclusion (TIDE) study revealed that patients in the high-risk group had significantly higher TIDE scores than those in the other groups (Fig. 8G) (*p* < 0.05).

## Discussion

Laryngeal cancer accounts for 25% of squamous cell carcinomas of the neck and head, yet there is still a lack of effective prognostic markers.^19^ In recent years, cuproptosis, a specific cell death mechanism, has been associated with the development of several solid tumors.^20^ Here, we used cuproptosis-related genes to construct a cuproptosis subtype that differs from previous clinical staging and could classify patients into different subgroups. There were significant differences found in the prognoses of patients with laryngeal carcinoma. In addition, a risk model constructed using cuproptosis-related genes that were significantly associated with laryngeal cancer patients performed well in the prediction of patient prognoses. This risk model was also a prognostic independent factor for patients with laryngeal cancer. Independent prognostic factors, including the risk model, Clinical_M, and Clinical_N, were used to construct a nomogram.

The risk model we constructed using cuproptosis-related genes included four genes. TMEM2 is a protein with a single-channel transmembrane domain protein.^21^ Other investigators have previously demonstrated that TMEM2 promoted developmental angiogenic Vegf signaling by regulating hyaluronic acid turnover.^22^ TMEM2 also promoted breast cancer cell metastasis,^23^ although no correlation between TMEM2 expression and laryngeal cancer has been reported as yet. In the present study, TMEM2 expression was a risk factor for laryngeal cancer. High expression of the DACT1 may have been invasive and metastatic in nasopharyngeal carcinoma cells.^24^ DACT1 is methylated in all oral squamous cell carcinomas.^25^ STMN2 encodes a stathmin family member of phosphoproteins and is upregulated in squamous cell carcinoma and lung adenocarcinoma.^26^ GPR173 is part of a highly conserved receptor family.^27^ In addition, no correlation between GPR173 and laryngeal cancer has been previously reported.

MYD1 is known as Signal Regulatory Protein Alpha (SIRPA) and was upregulated in the high-risk group. Furthermore, MYD1 was found to be a receptor for CD47 and was significantly upregulated in hot tumors.^28^ The binding of SIRPA to CD47 triggers inhibitory pathways in some cancers, ultimately leading to the immune escape of tumor cells.^29^ In addition, the phagocytic activity of macrophages and other phagocytes in tumors is enhanced when CD47-SIRPA interactions are blocked.^30^ The expression of 4-1BB in activated T-cells can cause a signaling cascade that leads to the upregulation of anti-apoptotic molecules. Targeting 4-1BB with agonist antibodies in tumor models can induce clear and durable antitumor immunity.^31^ Agonistic anti-human 4-1BB antibodies and second-/third-generation 4-1BB/CD3ζ Chimeric Antigen Receptor (CAR) T-cells have entered clinical trials.^32^ The two groups responded differently to numerous chemotherapy drugs. BX-795 is an inhibitor of 3-phosphoinositide-dependent kinase 1, which plays an active role in the treatment of many solid tumors.^33^ Different sensitivities to chemotherapeutic agents may also indicate different outcomes for patients in different risk groups receiving treatment.

GSEA revealed that high-risk samples showed upregulated expression of pathways involved in angiogenesis, Epithelial-Mesenchymal Transition (EMT), and KRAS signaling. Angiogenesis is the process by which new capillaries grow out of existing vessels, and abnormal angiogenesis is an important sign of cancer development.^34^ Studies have found increased vascularity and invasion in cases of head and neck tumors.^35^ Schlüter et al. found that high expression of angiogenic markers was associated with a high risk of squamous cell carcinoma of the larynx.^36^ Stimulation with bile acids and pepsin can promote carcinogenesis in the pharynx through EMT and is a risk factor for laryngeal cancer.^37^, ^38^ Aberrant KRAS expression leads to uncontrolled cell proliferation and ultimately moves the cells in a direction that favors metastasis.^39^ It has also been found that KRAS overexpression was associated with the progressive dedifferentiation of laryngeal squamous cell carcinoma.^40^

Our analysis primarily relies on retrospective data from specific databases, which may not represent the global diversity of laryngeal cancer cases. Additionally, the molecular mechanisms behind the observed associations with cuproptosis-related genes require further elucidation. While our findings offer promising avenues for future research and potential therapeutic targets, they need to be validated through prospective studies and integrated with other known biomarkers of laryngeal cancer.

## Conclusion

In conclusion, cuproptosis-related genes were used to subtype laryngeal cancer. The constructed cuproptosis risk model showed a good predictive performance for the prognosis of patients with laryngeal cancer. The risk model and nomogram produced here may be useful for guiding future clinical practice. This study also provides potential targets for laryngeal cancer therapy and a basis for studying the mechanisms by which cuproptosis plays a role in laryngeal cancer.

### Therapeutic analysis

We assessed patient sensitivity to chemotherapeutic agents using the Greedy Double Subspaces Coordinate Descent (GDSCD) (https://www.cancerrxgene.org/). To quantify IC50 values, the pRRophetic package^41^ in R was used, whilst the Wilcoxon test was used to investigate the differences in IC50 values among 138 chemotherapeutic agents across risk groups.

The TIDE database was used to assess patient responses to immune checkpoint therapy. For patients in different risk groups, we used the Wilcoxon test to compare their respective TIDE scores.

## Availability of data and materials

The datasets used and/or analyzed during the current study are available from the corresponding author on request.

## Authors’ contributions

Conceptualization, methodology, validation, writing-original draft preparation, writing-review, and editing, C.L.; Software and formal analysis, Y.Z.; Supervision, project administration, and funding acquisition, S.S. All authors reviewed the manuscript.

## Funding

No financial support was obtained.

## Conflicts of interest

The authors declare no conflicts of interest.

## References

[1] Lauwerends L.J., Galema H.A. (2021). Current intraoperative imaging techniques to improve surgical resection of laryngeal cancer: a systematic review. Cancers (Basel)..

[2] Obid R., Redlich M., Tomeh C. (2019). The treatment of laryngeal cancer. Oral Maxillofac Surg Clin North Am..

[3] Brandstorp-Boesen J., Sørum Falk R., Folkvard Evensen J., Boysen M., Brøndbo K. (2016). Risk of recurrence in laryngeal cancer. PloS One..

[4] Koroulakis A., Agarwal M. (2022).

[5] Tang D., Chen X., Kang R., Kroemer G. (2021). Ferroptosis: molecular mechanisms and health implications. Cell Res..

[6] Tsvetkov P., Coy S. (2022). Copper induces cell death by targeting lipoylated TCA cycle proteins. Science..

[7] Zhang G., Sun J., Zhang X. (2022). A novel Cuproptosis-related LncRNA signature to predict prognosis in hepatocellular carcinoma. Sci Rep..

[8] Yun Y., Wang Y., Yang E., Jing X. (2022). Cuproptosis-related gene - SLC31A1, FDX1 and ATP7B - polymorphisms are associated with risk of lung cancer. Pharmacogenomics Pers Med..

[9] Sha S., Si L., Wu X., Chen Y., Xiong H., Xu Y. (2022). Prognostic analysis of cuproptosis-related gene in triple-negative breast cancer. Front Immunol..

[10] Huang J., Xu Z., Yuan Z., Cheng L., Zhou C. (2022). Identification of cuproptosis-related subtypes and characterization of the tumor microenvironment landscape in head and neck squamous cell carcinoma. J Clin Lab Anal..

[11] Fountzilas E., Kotoula V., Angouridakis N., Karasmanis I., Wirtz R.M., Eleftheraki A.G. (2013). Identification and validation of a multigene predictor of recurrence in primary laryngeal cancer. PloS One..

[12] Wilkerson M.D., Hayes D.N. (2010). ConsensusClusterPlus: a class discovery tool with confidence assessments and item tracking. Bioinformatics..

[13] Hänzelmann S., Castelo R., Guinney J. (2013). GSVA: gene set variation analysis for microarray and RNA-seq data. BMC Bioinformatics..

[14] Rizvi A.A., Karaesmen E., Morgan M., Preus L., Wang J., Sovic M. (2019). gwasurvivr: an R package for genome-wide survival analysis. Bioinformatics..

[15] Chen B., Khodadoust M.S., Liu C.L., Newman A.M., Alizadeh A.A. (2018). Profiling tumor infiltrating immune cells with CIBERSORT. Methods Mol Biol..

[16] Hu D., Zhou M., Zhu X. (2019). Deciphering immune-associated genes to predict survival in clear cell renal cell cancer. Biomed Res Int..

[17] Ritchie M.E., Phipson B., Wu D., Hu Y., Law C.W., Shi W. (2015). limma powers differential expression analyses for RNA-sequencing and microarray studies. Nucleic Acids Res..

[18] Zhang S., Tong Y.X., Zhang X.H., Zhang Y.J., Xu X.S., Xiao A.T. (2019). A novel and validated nomogram to predict overall survival for gastric neuroendocrine neoplasms. J Cancer..

[19] Elicin O., Giger R. (2020). Comparison of current surgical and non-surgical treatment strategies for early and locally advanced stage glottic laryngeal cancer and their outcome. Cancers (Basel)..

[20] Huang X., Zhou S., Tóth J., Hajdu A. (2022). Cuproptosis-related gene index: a predictor for pancreatic cancer prognosis, immunotherapy efficacy, and chemosensitivity. Front Immunol..

[21] Yoshida H., Nagaoka A., Kusaka-Kikushima A., Tobiishi M., Kawabata K., Sayo T. (2013). KIAA1199, a deafness gene of unknown function, is a new hyaluronan binding protein involved in hyaluronan depolymerization. Proc Natl Acad Sci USA..

[22] De Angelis J.E., Lagendijk A.K., Chen H., Tromp A., Bower N.I., Tunny K.A. (2017). Tmem2 regulates embryonic Vegf signaling by controlling hyaluronic acid turnover. Dev Cell..

[23] Lee H., Goodarzi H., Tavazoie S.F., Alarcón C.R. (2016). TMEM2 is a SOX4-regulated gene that mediates metastatic migration and invasion in breast cancer. Cancer Res..

[24] Yang J.H., Lin L.K., Zhang S. (2019). Effects of DACT1 methylation status on invasion and metastasis of nasopharyngeal carcinoma. Biol Res..

[25] Paluszczak J., Wiśniewska D., Kostrzewska-Poczekaj M., Kiwerska K., Grénman R., Mielcarek-Kuchta D. (2017). Prognostic significance of the methylation of Wnt pathway antagonists-CXXC4, DACT2, and the inhibitors of sonic hedgehog signaling-ZIC1, ZIC4, and HHIP in head and neck squamous cell carcinomas. Clin Oral Investig..

[26] Zhang Y., He R.-Q., Dang Y.-W., Zhang X.-L., Wang X., Huang S.-N. (2016). Comprehensive analysis of the long noncoding RNA HOXA11-AS gene interaction regulatory network in NSCLC cells. Cancer Cell Int..

[27] Treen A.K., Luo V., Belsham D.D. (2016). Phoenixin activates immortalized GnRH and Kisspeptin neurons through the novel receptor GPR173. Mol Endocrinol..

[28] Barclay A.N., Van den Berg T.K. (2014). The interaction between signal regulatory protein alpha (SIRPα) and CD47: structure, function, and therapeutic target. Annu Rev Immunol..

[29] Qiang H., Li J., Chang Q., Shen Y., Qian J., Chu T. (2022). Mining GEO and TCGA database for immune microenvironment of lung squamous cell carcinoma patients with or without chemotherapy. Front Oncol..

[30] Lu Q., Chen X., Wang S., Lu Y., Yang C. (2020). Potential new cancer immunotherapy: anti-CD47-SIRPα antibodies. Onco Targets Ther..

[31] Chester C., Sanmamed M.F., Wang J., Melero I. (2018). Immunotherapy targeting 4-1BB: mechanistic rationale, clinical results, and future strategies. Blood..

[32] Claus C., Ferrara C., Xu W., Sam J., Lang S., Uhlenbrock F. (2019). Tumor-targeted 4-1BB agonists for combination with T cell bispecific antibodies as off-the-shelf therapy. Sci Transl Med..

[33] Choi E.A., Choi Y.S., Lee E.J., Singh S.R., Kim S.C., Chang S. (2019). A pharmacogenomic analysis using L1000CDS(2) identifies BX-795 as a potential anticancer drug for primary pancreatic ductal adenocarcinoma cells. Cancer Lett..

[34] Li T., Kang G., Wang T., Huang H. (2018). Tumor angiogenesis and anti-angiogenic gene therapy for cancer. Oncol Lett..

[35] Sion-Vardy N., Fliss D.M., Prinsloo I., Shoham-Vardi I., Benharroch D. (2001). Neoangiogenesis in squamous cell carcinoma of the larynx - biological and prognostic associations. Pathol Res Pract..

[36] Schlüter A., Weller P., Kanaan O., Nel I., Heusgen L., Höing B. (2018). CD31 and VEGF are prognostic biomarkers in early-stage, but not in late-stage, laryngeal squamous cell carcinoma. BMC Cancer..

[37] Shellman Z., Aldhahrani A., Verdon B., Mather M., Paleri V. (2017). Bile acids: a potential role in the pathogenesis of pharyngeal malignancy. Clin Otolaryngol..

[38] Tan J.J., Wang L., Mo T.T., Wang J., Wang M.G., Li X.P. (2019). Pepsin promotes IL-8 signaling-induced epithelial-mesenchymal transition in laryngeal carcinoma. Cancer Cell Int..

[39] Ferrer I., Zugazagoitia J., Herbertz S., John W., Paz-Ares L., Schmid-Bindert G. (2018). KRAS-Mutant non-small cell lung cancer: from biology to therapy. Lung Cancer..

[40] Papanikolaou V., Chrysovergis A., Mastronikolis S., Tsiambas E., Ragos V., Peschos D. (2021). Impact of K-Ras over-expression in laryngeal squamous cell carcinoma. In vivo (Athens, Greece). In Vivo..

[41] Geeleher P., Cox N., Huang R.S. (2014). pRRophetic: an R package for prediction of clinical chemotherapeutic response from tumor gene expression levels. PloS One..

