# Supplementary material for: Effective prognostic risk model with cuproptosis-related genes in laryngeal cancer
Source: Braz J Otorhinolaryngol. 2023 Dec 26;90(2):101384. doi: 10.1016/j.bjorl.2023.101384 (PMC10823110; doi:10.1016/j.bjorl.2023.101384)

**BJORL-D-23-00237_Supplementary Material**

**Supplementary Table 1** Differentially expressed genes between C1 and C2 subtypes.

| **Gene** | **log2FC** | **AveExpr** | **t** | **p-value** | **adj.P.Val** | **B** | **Regulated** |
| --- | --- | --- | --- | --- | --- | --- | --- |
| CHGB | 5.228607 | 4.751762 | 8.719072 | 3.10E-13 | 6.36E-09 | 19.47847 | Up-Regulated |
| LMOD3 | 3.680587 | 2.716105 | 7.438875 | 1.00E-10 | 1.03E-06 | 14.06557 | Up-Regulated |
| MYF6 | 3.652043 | 1.883542 | 7.228241 | 2.56E-10 | 1.75E-06 | 13.18252 | Up-Regulated |
| MYOT | 3.771031 | 3.066116 | 7.124708 | 4.06E-10 | 1.88E-06 | 12.74998 | Up-Regulated |
| SMPX | 3.7141 | 2.399481 | 7.097267 | 4.58E-10 | 1.88E-06 | 12.63552 | Up-Regulated |
| MYH1 | 5.5822 | 3.214048 | 7.035551 | 6.03E-10 | 2.06E-06 | 12.37842 | Up-Regulated |
| MYH8 | 3.564494 | 1.804356 | 6.959806 | 8.43E-10 | 2.21E-06 | 12.06346 | Up-Regulated |
| CACNA1S | 3.79127 | 2.116086 | 6.946159 | 8.95E-10 | 2.21E-06 | 12.00679 | Up-Regulated |
| DYSFIP1 | 3.127507 | 2.664554 | 6.928057 | 9.70E-10 | 2.21E-06 | 11.93165 | Up-Regulated |
| MYL2 | 4.557856 | 3.012352 | 6.884937 | 1.17E-09 | 2.26E-06 | 11.75284 | Up-Regulated |
| CASQ2 | 3.633996 | 4.130532 | 6.846182 | 1.39E-09 | 2.26E-06 | 11.59233 | Up-Regulated |
| MYBPH | 4.704476 | 3.178379 | 6.845247 | 1.40E-09 | 2.26E-06 | 11.58846 | Up-Regulated |
| SMYD1 | 3.587311 | 1.814233 | 6.840239 | 1.43E-09 | 2.26E-06 | 11.56774 | Up-Regulated |
| MYL1 | 5.043069 | 2.884132 | 6.808674 | 1.64E-09 | 2.29E-06 | 11.43719 | Up-Regulated |
| KBTBD10 | 4.174439 | 4.449607 | 6.790242 | 1.78E-09 | 2.29E-06 | 11.36101 | Up-Regulated |
| LMOD2 | 3.839024 | 2.036465 | 6.78999 | 1.78E-09 | 2.29E-06 | 11.35997 | Up-Regulated |
| MYPN | 3.598756 | 2.530637 | 6.751296 | 2.11E-09 | 2.41E-06 | 11.20023 | Up-Regulated |
| CHRND | 3.32105 | 1.535285 | 6.742197 | 2.20E-09 | 2.41E-06 | 11.1627 | Up-Regulated |
| ASB5 | 3.781974 | 2.081647 | 6.725216 | 2.37E-09 | 2.41E-06 | 11.09268 | Up-Regulated |
| MYLPF | 3.943056 | 3.139607 | 6.717856 | 2.45E-09 | 2.41E-06 | 11.06235 | Up-Regulated |
| CHRNA1 | 3.486774 | 3.495025 | 6.716065 | 2.47E-09 | 2.41E-06 | 11.05496 | Up-Regulated |
| CSRP3 | 4.514835 | 2.487991 | 6.678993 | 2.90E-09 | 2.69E-06 | 10.90231 | Up-Regulated |
| ANKRD1 | 3.689069 | 3.551543 | 6.670328 | 3.01E-09 | 2.69E-06 | 10.86666 | Up-Regulated |
| TMEM8C | 1.475474 | 0.623217 | 6.632046 | 3.56E-09 | 2.95E-06 | 10.70929 | Up-Regulated |
| ACTC1 | 5.273313 | 4.420036 | 6.630349 | 3.59E-09 | 2.95E-06 | 10.70232 | Up-Regulated |
| MYOG | 3.811026 | 2.201012 | 6.603013 | 4.04E-09 | 3.03E-06 | 10.5901 | Up-Regulated |
| TTN | 3.106691 | 7.827365 | 6.599649 | 4.10E-09 | 3.03E-06 | 10.5763 | Up-Regulated |
| ATP1B4 | 2.61595 | 1.234526 | 6.598139 | 4.13E-09 | 3.03E-06 | 10.57011 | Up-Regulated |
| MYL4 | 2.044554 | 2.17539 | 6.577309 | 4.52E-09 | 3.20E-06 | 10.48469 | Up-Regulated |
| CHRNG | 2.923735 | 1.875088 | 6.547741 | 5.15E-09 | 3.52E-06 | 10.36356 | Up-Regulated |
| NRAP | 4.866528 | 3.019352 | 6.502706 | 6.26E-09 | 3.89E-06 | 10.17936 | Up-Regulated |
| ACTN2 | 4.483348 | 3.939064 | 6.501644 | 6.29E-09 | 3.89E-06 | 10.17502 | Up-Regulated |
| PRKAG3 | 2.730398 | 1.852249 | 6.501453 | 6.30E-09 | 3.89E-06 | 10.17424 | Up-Regulated |
| DUSP27 | 3.318574 | 2.270491 | 6.49593 | 6.45E-09 | 3.89E-06 | 10.15167 | Up-Regulated |
| CAV3 | 2.847467 | 1.370374 | 6.485942 | 6.74E-09 | 3.95E-06 | 10.11088 | Up-Regulated |
| ARPP21 | 3.087128 | 1.690478 | 6.456508 | 7.65E-09 | 4.37E-06 | 9.990754 | Up-Regulated |
| MYH7 | 4.541133 | 3.490322 | 6.439088 | 8.26E-09 | 4.58E-06 | 9.919736 | Up-Regulated |
| ANKRD23 | 2.220987 | 5.741398 | 6.428214 | 8.65E-09 | 4.68E-06 | 9.875434 | Up-Regulated |
| HRC | 2.75863 | 3.771343 | 6.40432 | 9.60E-09 | 5.05E-06 | 9.778154 | Up-Regulated |
| MYF5 | 1.913663 | 0.814821 | 6.391751 | 1.01E-08 | 5.20E-06 | 9.727028 | Up-Regulated |
| CACNG1 | 2.919235 | 1.804999 | 6.328914 | 1.33E-08 | 6.66E-06 | 9.471856 | Up-Regulated |
| TNNI1 | 2.623674 | 7.061232 | 6.311196 | 1.44E-08 | 7.02E-06 | 9.400044 | Up-Regulated |
| HHATL | 2.70437 | 1.406838 | 6.297211 | 1.52E-08 | 7.28E-06 | 9.343405 | Up-Regulated |
| VGLL2 | 1.966313 | 1.217788 | 6.28545 | 1.60E-08 | 7.49E-06 | 9.2958 | Up-Regulated |
| APOBEC2 | 2.952398 | 2.415323 | 6.272774 | 1.69E-08 | 7.73E-06 | 9.244523 | Up-Regulated |
| MYBPC2 | 4.154198 | 4.12972 | 6.255846 | 1.82E-08 | 7.98E-06 | 9.176095 | Up-Regulated |
| MYOZ2 | 3.004019 | 1.979902 | 6.255239 | 1.83E-08 | 7.98E-06 | 9.173643 | Up-Regulated |
| XIRP2 | 4.338959 | 2.78536 | 6.241411 | 1.94E-08 | 8.29E-06 | 9.117793 | Up-Regulated |
| ITGB1BP3 | 2.900333 | 1.3178 | 6.233089 | 2.01E-08 | 8.33E-06 | 9.084198 | Up-Regulated |
| MURC | 2.328004 | 4.384749 | 6.230944 | 2.03E-08 | 8.33E-06 | 9.075544 | Up-Regulated |
| MYH3 | 2.710054 | 5.916616 | 6.1944 | 2.37E-08 | 9.55E-06 | 8.928206 | Up-Regulated |
| ACTA1 | 4.807626 | 4.716527 | 6.180297 | 2.52E-08 | 9.95E-06 | 8.87142 | Up-Regulated |
| KBTBD5 | 3.00452 | 1.536735 | 6.12768 | 3.16E-08 | 1.20E-05 | 8.659927 | Up-Regulated |
| A2BP1 | 2.292115 | 1.55076 | 6.124137 | 3.21E-08 | 1.20E-05 | 8.645709 | Up-Regulated |
| HFE2 | 2.666839 | 1.3915 | 6.122577 | 3.23E-08 | 1.20E-05 | 8.639449 | Up-Regulated |
| COX6A2 | 3.262985 | 1.809732 | 6.118484 | 3.28E-08 | 1.20E-05 | 8.623026 | Up-Regulated |
| MYOD1 | 2.28977 | 1.339179 | 6.093844 | 3.65E-08 | 1.31E-05 | 8.52424 | Up-Regulated |
| BEST3 | 2.768144 | 2.221944 | 6.035687 | 4.68E-08 | 1.65E-05 | 8.291613 | Up-Regulated |
| TNNC1 | 3.547174 | 4.191825 | 6.022422 | 4.95E-08 | 1.72E-05 | 8.238663 | Up-Regulated |
| C10orf71 | 2.72512 | 1.45946 | 5.993194 | 5.60E-08 | 1.92E-05 | 8.12213 | Up-Regulated |
| MYH2 | 5.21112 | 3.591846 | 5.982076 | 5.87E-08 | 1.98E-05 | 8.077853 | Up-Regulated |
| CACNB1 | 1.269946 | 7.226369 | 5.969862 | 6.18E-08 | 2.05E-05 | 8.029245 | Up-Regulated |
| ASB10 | 1.70917 | 0.751372 | 5.954618 | 6.60E-08 | 2.15E-05 | 7.96863 | Up-Regulated |
| ATP1A2 | 2.957756 | 4.401063 | 5.886218 | 8.81E-08 | 2.83E-05 | 7.697336 | Up-Regulated |
| PPP1R3A | 2.210422 | 1.053226 | 5.875261 | 9.22E-08 | 2.91E-05 | 7.653981 | Up-Regulated |
| SLN | 3.470285 | 3.487873 | 5.857751 | 9.93E-08 | 3.07E-05 | 7.584762 | Up-Regulated |
| ASB15 | 1.386344 | 0.673411 | 5.853241 | 1.01E-07 | 3.07E-05 | 7.566945 | Up-Regulated |
| CA3 | 2.775194 | 3.355237 | 5.852354 | 1.02E-07 | 3.07E-05 | 7.563444 | Up-Regulated |
| SCN4A | 2.814159 | 2.748183 | 5.840733 | 1.07E-07 | 3.17E-05 | 7.517563 | Up-Regulated |
| DES | 4.530176 | 6.204523 | 5.818147 | 1.17E-07 | 3.44E-05 | 7.428488 | Up-Regulated |
| TRDN | 3.763217 | 3.336337 | 5.813534 | 1.20E-07 | 3.46E-05 | 7.410312 | Up-Regulated |
| MYH4 | 2.281126 | 1.115923 | 5.683346 | 2.06E-07 | 5.88E-05 | 6.899626 | Up-Regulated |
| TNNI2 | 2.71805 | 5.600267 | 5.645056 | 2.42E-07 | 6.80E-05 | 6.750296 | Up-Regulated |
| NCAM1 | 2.48593 | 5.431862 | 5.638723 | 2.48E-07 | 6.81E-05 | 6.725637 | Up-Regulated |
| KBTBD12 | 2.048213 | 1.733017 | 5.638233 | 2.49E-07 | 6.81E-05 | 6.723729 | Up-Regulated |
| TCAP | 3.734617 | 4.237663 | 5.633344 | 2.54E-07 | 6.85E-05 | 6.7047 | Up-Regulated |
| CKM | 3.979776 | 5.219386 | 5.630559 | 2.57E-07 | 6.85E-05 | 6.693865 | Up-Regulated |
| SCN5A | 2.42473 | 3.783506 | 5.618614 | 2.70E-07 | 7.10E-05 | 6.647412 | Up-Regulated |
| C20orf166 | 1.877535 | 0.80891 | 5.606727 | 2.84E-07 | 7.37E-05 | 6.601222 | Up-Regulated |
| PRUNE2 | 2.303959 | 6.225323 | 5.543308 | 3.69E-07 | 9.46E-05 | 6.355487 | Up-Regulated |
| TMOD4 | 2.357798 | 2.302827 | 5.532178 | 3.86E-07 | 9.78E-05 | 6.312479 | Up-Regulated |
| ABRA | 1.652024 | 1.04351 | 5.502959 | 4.35E-07 | 0.000109 | 6.19975 | Up-Regulated |
| XIRP1 | 2.994131 | 4.882894 | 5.489625 | 4.60E-07 | 0.000114 | 6.148394 | Up-Regulated |
| CMYA5 | 2.32105 | 6.690478 | 5.402494 | 6.57E-07 | 0.000161 | 5.814114 | Up-Regulated |
| ENO3 | 2.110711 | 5.807263 | 5.368895 | 7.54E-07 | 0.000182 | 5.68584 | Up-Regulated |
| MYL3 | 2.356828 | 2.113604 | 5.366438 | 7.61E-07 | 0.000182 | 5.676476 | Up-Regulated |
| MYOZ1 | 2.805157 | 3.75811 | 5.354311 | 8.00E-07 | 0.000189 | 5.630275 | Up-Regulated |
| MYLK2 | 1.977222 | 3.244907 | 5.322373 | 9.11E-07 | 0.000212 | 5.50882 | Up-Regulated |
| ATP2A1 | 2.497919 | 4.765921 | 5.316371 | 9.33E-07 | 0.000215 | 5.486035 | Up-Regulated |
| PYGM | 2.3637 | 3.830663 | 5.301797 | 9.90E-07 | 0.000226 | 5.430751 | Up-Regulated |
| MYH6 | 2.631741 | 1.933188 | 5.264253 | 1.15E-06 | 0.00026 | 5.288657 | Up-Regulated |
| MYOZ3 | 1.976893 | 3.28979 | 5.235559 | 1.29E-06 | 0.000289 | 5.180372 | Up-Regulated |
| MYADML2 | 1.952774 | 1.915588 | 5.196114 | 1.52E-06 | 0.000335 | 5.03197 | Up-Regulated |
| LRRC14B | 1.371809 | 1.28646 | 5.133039 | 1.95E-06 | 0.000421 | 4.795771 | Up-Regulated |
| SGCG | 2.132643 | 2.601146 | 5.132491 | 1.96E-06 | 0.000421 | 4.793724 | Up-Regulated |
| HSPB7 | 2.79152 | 5.167342 | 5.130716 | 1.97E-06 | 0.000421 | 4.787099 | Up-Regulated |
| TNNC2 | 2.860894 | 4.325211 | 5.069873 | 2.51E-06 | 0.000532 | 4.56063 | Up-Regulated |
| FSD2 | 1.042113 | 0.651899 | 5.053441 | 2.68E-06 | 0.000562 | 4.499696 | Up-Regulated |
| MUSTN1 | 2.003533 | 4.572093 | 5.046028 | 2.76E-06 | 0.000573 | 4.472239 | Up-Regulated |
| RBM24 | 2.21215 | 3.807774 | 5.037947 | 2.85E-06 | 0.000585 | 4.442329 | Up-Regulated |
| TRIM63 | 2.7465 | 2.855174 | 5.023487 | 3.02E-06 | 0.000614 | 4.388867 | Up-Regulated |
| STAC3 | 1.942619 | 6.105554 | 5.005157 | 3.25E-06 | 0.000653 | 4.321212 | Up-Regulated |
| CASQ1 | 2.334278 | 2.786537 | 4.990598 | 3.44E-06 | 0.000685 | 4.267563 | Up-Regulated |
| NPY6R | 1.347424 | 1.021888 | 4.975489 | 3.65E-06 | 0.000718 | 4.211969 | Up-Regulated |
| LRRC2 | 1.950922 | 3.048815 | 4.973829 | 3.67E-06 | 0.000718 | 4.205868 | Up-Regulated |
| SHOX | 1.253233 | 0.709448 | 4.954824 | 3.96E-06 | 0.000767 | 4.136074 | Up-Regulated |
| YIPF7 | 1.23985 | 0.7481 | 4.921417 | 4.51E-06 | 0.000866 | 4.013721 | Up-Regulated |
| NOG | 1.801663 | 1.901354 | 4.890379 | 5.10E-06 | 0.000969 | 3.900426 | Up-Regulated |
| LDB3 | 2.190156 | 4.439637 | 4.867313 | 5.58E-06 | 0.00105 | 3.816473 | Up-Regulated |
| NEB | 2.650161 | 7.754241 | 4.856522 | 5.82E-06 | 0.001086 | 3.777268 | Up-Regulated |
| ALPK3 | 1.348211 | 7.409878 | 4.854032 | 5.87E-06 | 0.001086 | 3.768228 | Up-Regulated |
| SYPL2 | 1.861315 | 3.553246 | 4.799719 | 7.25E-06 | 0.00133 | 3.571658 | Up-Regulated |
| S100A1 | 1.839017 | 5.50807 | 4.782488 | 7.75E-06 | 0.001409 | 3.50954 | Up-Regulated |
| LRRC39 | 1.637098 | 2.630168 | 4.773355 | 8.03E-06 | 0.001447 | 3.476665 | Up-Regulated |
| C8orf22 | 1.027852 | 0.456488 | 4.758328 | 8.51E-06 | 0.00152 | 3.422644 | Up-Regulated |
| FBXO40 | 1.485037 | 1.142738 | 4.743176 | 9.02E-06 | 0.001597 | 3.368272 | Up-Regulated |
| EXTL1 | 1.242098 | 1.590357 | 4.729321 | 9.52E-06 | 0.00167 | 3.318635 | Up-Regulated |
| ASB16 | 1.071948 | 3.475883 | 4.724172 | 9.71E-06 | 0.001689 | 3.300207 | Up-Regulated |
| DDN | 1.699057 | 5.503347 | 4.701663 | 1.06E-05 | 0.001826 | 3.219782 | Up-Regulated |
| UNC45B | 2.702369 | 3.265177 | 4.683858 | 1.13E-05 | 0.001939 | 3.156313 | Up-Regulated |
| MYOM1 | 1.770317 | 4.8603 | 4.674616 | 1.17E-05 | 0.001992 | 3.12342 | Up-Regulated |
| MYBPC1 | 4.068298 | 4.334116 | 4.666923 | 1.21E-05 | 0.002035 | 3.096071 | Up-Regulated |
| DDIT4L | 2.13312 | 4.502964 | 4.65989 | 1.24E-05 | 0.002073 | 3.071085 | Up-Regulated |
| TMEM233 | 1.11322 | 1.963631 | 4.64563 | 1.31E-05 | 0.002172 | 3.020495 | Up-Regulated |
| RAPSN | 2.090848 | 2.838023 | 4.635765 | 1.36E-05 | 0.002225 | 2.985545 | Up-Regulated |
| DUSP26 | 2.142572 | 3.261311 | 4.635092 | 1.37E-05 | 0.002225 | 2.983163 | Up-Regulated |
| DGKD | 0.635631 | 8.858709 | 4.500975 | 2.27E-05 | 0.003665 | 2.512252 | Up-Regulated |
| TNNT3 | 2.765506 | 6.2976 | 4.467133 | 2.57E-05 | 0.004127 | 2.394683 | Up-Regulated |
| PALM2 | 1.310017 | 4.741407 | 4.460467 | 2.64E-05 | 0.004199 | 2.371588 | Up-Regulated |
| C2CD4B | 1.137844 | 3.661356 | 4.389005 | 3.44E-05 | 0.005393 | 2.125258 | Up-Regulated |
| CKMT2 | 2.208413 | 3.24568 | 4.382244 | 3.53E-05 | 0.005488 | 2.102076 | Up-Regulated |
| CXorf64 | 1.233339 | 0.702359 | 4.378605 | 3.58E-05 | 0.005521 | 2.089607 | Up-Regulated |
| CDH15 | 1.748926 | 3.256983 | 4.370528 | 3.68E-05 | 0.005645 | 2.061955 | Up-Regulated |
| FLNC | 1.630402 | 8.986062 | 4.328152 | 4.31E-05 | 0.006551 | 1.917373 | Up-Regulated |
| MUSK | 1.467978 | 1.470533 | 4.325363 | 4.35E-05 | 0.00657 | 1.907887 | Up-Regulated |
| PTPN5 | 1.450954 | 2.423057 | 4.320626 | 4.43E-05 | 0.006636 | 1.891783 | Up-Regulated |
| SPTB | 1.233876 | 7.59709 | 4.306912 | 4.66E-05 | 0.006926 | 1.845226 | Up-Regulated |
| SGCD | 1.707852 | 6.32418 | 4.305025 | 4.69E-05 | 0.006926 | 1.838826 | Up-Regulated |
| ITIH4 | 1.55103 | 5.068888 | 4.301974 | 4.74E-05 | 0.006954 | 1.828483 | Up-Regulated |
| ASB11 | 0.830259 | 0.380016 | 4.296335 | 4.84E-05 | 0.007048 | 1.809379 | Up-Regulated |
| FITM1 | 1.253743 | 3.02702 | 4.294152 | 4.88E-05 | 0.007055 | 1.801986 | Up-Regulated |
| SCG2 | 1.661078 | 5.504211 | 4.292057 | 4.92E-05 | 0.00706 | 1.794895 | Up-Regulated |
| CFL2 | 0.845737 | 8.127975 | 4.262911 | 5.47E-05 | 0.007795 | 1.696453 | Up-Regulated |
| ANO5 | 1.70965 | 3.166315 | 4.26111 | 5.51E-05 | 0.007795 | 1.690384 | Up-Regulated |
| MYLK3 | 1.422343 | 2.548257 | 4.233853 | 6.08E-05 | 0.008549 | 1.59872 | Up-Regulated |
| PLN | 1.515937 | 3.713131 | 4.225393 | 6.27E-05 | 0.0087 | 1.570344 | Up-Regulated |
| ADAMTS5 | 1.28167 | 6.308172 | 4.225283 | 6.27E-05 | 0.0087 | 1.569975 | Up-Regulated |
| PHKG1 | 1.398902 | 2.661373 | 4.210559 | 6.62E-05 | 0.009061 | 1.520672 | Up-Regulated |
| KLHL30 | 1.660389 | 3.655922 | 4.210356 | 6.62E-05 | 0.009061 | 1.519993 | Up-Regulated |
| ITGB1BP2 | 1.072743 | 2.846194 | 4.207795 | 6.68E-05 | 0.009085 | 1.511429 | Up-Regulated |
| AKAP6 | 1.531126 | 6.266735 | 4.164942 | 7.80E-05 | 0.010466 | 1.368609 | Up-Regulated |
| MYOM2 | 1.817141 | 4.865499 | 4.149505 | 8.25E-05 | 0.010992 | 1.317385 | Up-Regulated |
| CACNG6 | 1.749026 | 1.89939 | 4.139205 | 8.56E-05 | 0.011271 | 1.283275 | Up-Regulated |
| IP6K3 | 2.135239 | 3.50653 | 4.138913 | 8.56E-05 | 0.011271 | 1.282308 | Up-Regulated |
| ADAMTS6 | 1.196965 | 4.864675 | 4.128662 | 8.89E-05 | 0.011619 | 1.248415 | Up-Regulated |
| SGCA | 1.821567 | 3.599115 | 4.072146 | 0.000109 | 0.014036 | 1.062515 | Up-Regulated |
| BMP5 | 1.421435 | 1.143225 | 4.065114 | 0.000111 | 0.014301 | 1.0395 | Up-Regulated |
| KLHL31 | 1.252233 | 4.550941 | 4.061775 | 0.000113 | 0.014382 | 1.028579 | Up-Regulated |
| ART1 | 0.680278 | 0.428922 | 4.05335 | 0.000116 | 0.014726 | 1.001052 | Up-Regulated |
| PPP1R1A | 1.500454 | 1.566749 | 4.04168 | 0.000121 | 0.015254 | 0.962983 | Up-Regulated |
| SMTNL2 | 1.710172 | 3.389785 | 4.023291 | 0.000129 | 0.016179 | 0.903144 | Up-Regulated |
| MEF2C | 1.14952 | 7.826494 | 4.012987 | 0.000134 | 0.016676 | 0.869691 | Up-Regulated |
| ADCY2 | 1.824543 | 4.328009 | 4.001106 | 0.00014 | 0.017284 | 0.831184 | Up-Regulated |
| NNAT | 1.303507 | 3.757491 | 3.992004 | 0.000144 | 0.017739 | 0.801738 | Up-Regulated |
| PDLIM3 | 1.795148 | 8.078783 | 3.980057 | 0.00015 | 0.018389 | 0.763155 | Up-Regulated |
| SLC30A2 | 0.925939 | 1.203919 | 3.977001 | 0.000152 | 0.018477 | 0.753297 | Up-Regulated |
| MB | 2.293796 | 6.139202 | 3.971421 | 0.000155 | 0.018732 | 0.735309 | Up-Regulated |
| ADD2 | 2.238585 | 5.259928 | 3.969032 | 0.000156 | 0.018779 | 0.727612 | Up-Regulated |
| PGAM2 | 1.182759 | 5.21876 | 3.960482 | 0.000161 | 0.019237 | 0.700096 | Up-Regulated |
| TMEM2 | 0.816893 | 10.13439 | 3.940831 | 0.000173 | 0.020485 | 0.637001 | Up-Regulated |
| FHL1 | 1.52632 | 9.010164 | 3.935959 | 0.000176 | 0.020716 | 0.62139 | Up-Regulated |
| KCND2 | 1.329802 | 4.123732 | 3.917251 | 0.000187 | 0.021983 | 0.561565 | Up-Regulated |
| CCL7 | 1.376409 | 2.476953 | 3.910974 | 0.000192 | 0.022217 | 0.541534 | Up-Regulated |
| RGS16 | 0.904609 | 7.656416 | 3.910941 | 0.000192 | 0.022217 | 0.54143 | Up-Regulated |
| DCLK1 | 1.698128 | 5.836711 | 3.908277 | 0.000193 | 0.022297 | 0.532935 | Up-Regulated |
| KLHL34 | 1.221887 | 1.262683 | 3.906211 | 0.000195 | 0.022332 | 0.52635 | Up-Regulated |
| ANGPTL7 | 1.2775 | 1.503615 | 3.897754 | 0.000201 | 0.022869 | 0.499418 | Up-Regulated |
| KISS1 | 0.88378 | 0.936665 | 3.894355 | 0.000203 | 0.023013 | 0.488604 | Up-Regulated |
| PCDH9 | 1.399828 | 2.835737 | 3.891864 | 0.000205 | 0.023085 | 0.480685 | Up-Regulated |
| PDE4DIP | 0.850256 | 10.16292 | 3.858655 | 0.00023 | 0.025749 | 0.375416 | Up-Regulated |
| PVALB | 1.367389 | 1.726719 | 3.841144 | 0.000244 | 0.0272 | 0.320156 | Up-Regulated |
| EBF2 | 1.164072 | 2.255704 | 3.833996 | 0.00025 | 0.027726 | 0.297646 | Up-Regulated |
| B3GALT2 | 1.0949 | 2.421385 | 3.826657 | 0.000256 | 0.02828 | 0.274563 | Up-Regulated |
| STC1 | 0.996565 | 9.06832 | 3.818842 | 0.000263 | 0.028892 | 0.25002 | Up-Regulated |
| MAP1A | 1.267552 | 8.266451 | 3.804288 | 0.000277 | 0.030206 | 0.204398 | Up-Regulated |
| PPFIA2 | 0.997978 | 1.863222 | 3.792662 | 0.000288 | 0.031263 | 0.168044 | Up-Regulated |
| SORBS1 | 1.04815 | 7.13557 | 3.79013 | 0.00029 | 0.031332 | 0.160134 | Up-Regulated |
| DACT1 | 1.562044 | 6.567767 | 3.78892 | 0.000292 | 0.031332 | 0.156358 | Up-Regulated |
| GPR173 | 1.113607 | 2.913188 | 3.787155 | 0.000293 | 0.031357 | 0.150846 | Up-Regulated |
| MOBP | 1.39987 | 1.395083 | 3.774556 | 0.000306 | 0.032395 | 0.111572 | Up-Regulated |
| MAPT | 1.298744 | 4.865385 | 3.765809 | 0.000315 | 0.033201 | 0.084358 | Up-Regulated |
| ALDH1A2 | 1.281606 | 2.922611 | 3.723207 | 0.000364 | 0.037963 | -0.04756 | Up-Regulated |
| TMEM170B | 0.985967 | 5.588411 | 3.718301 | 0.00037 | 0.038401 | -0.06269 | Up-Regulated |
| GRPR | 1.111063 | 1.627077 | 3.71535 | 0.000374 | 0.03859 | -0.07178 | Up-Regulated |
| PRKAA2 | 1.660126 | 5.456664 | 3.709662 | 0.000381 | 0.03914 | -0.08929 | Up-Regulated |
| MATN3 | 1.393472 | 5.276559 | 3.704411 | 0.000388 | 0.03964 | -0.10544 | Up-Regulated |
| TIE1 | 0.705276 | 8.237523 | 3.698783 | 0.000396 | 0.040153 | -0.12273 | Up-Regulated |
| DOK5 | 1.105143 | 3.966301 | 3.695478 | 0.0004 | 0.040153 | -0.13287 | Up-Regulated |
| PADI2 | 1.29885 | 7.013281 | 3.694721 | 0.000401 | 0.040153 | -0.13519 | Up-Regulated |
| ASB12 | 0.934256 | 1.225163 | 3.684858 | 0.000414 | 0.041182 | -0.16542 | Up-Regulated |
| IL1R1 | 0.728478 | 10.39803 | 3.682324 | 0.000418 | 0.041255 | -0.17318 | Up-Regulated |
| DUPD1 | 0.659487 | 0.34852 | 3.677883 | 0.000424 | 0.041474 | -0.18677 | Up-Regulated |
| STMN2 | 1.000259 | 1.335723 | 3.661108 | 0.000449 | 0.04366 | -0.23799 | Up-Regulated |
| HAS2 | 1.371302 | 6.734428 | 3.6513 | 0.000464 | 0.0449 | -0.26786 | Up-Regulated |
| LIF | 1.3134 | 7.412 | 3.643868 | 0.000475 | 0.04581 | -0.29046 | Up-Regulated |
| DMPK | 0.67548 | 9.034384 | 3.641027 | 0.00048 | 0.046029 | -0.29909 | Up-Regulated |
| ST6GAL2 | 1.792793 | 4.396649 | 3.638223 | 0.000484 | 0.046245 | -0.3076 | Up-Regulated |
| CAMK2A | 1.537046 | 3.514243 | 3.629149 | 0.000499 | 0.047441 | -0.33512 | Up-Regulated |
| TRIM55 | 1.449413 | 2.403014 | 3.627249 | 0.000502 | 0.047521 | -0.34087 | Up-Regulated |
| TGFB3 | 0.955348 | 9.186842 | 3.620039 | 0.000514 | 0.048449 | -0.36269 | Up-Regulated |
| GADL1 | 0.649235 | 0.458951 | 3.61672 | 0.00052 | 0.048761 | -0.37273 | Up-Regulated |
| TPRG1L | -0.66684 | 10.91713 | -4.41757 | 3.10E-05 | 0.004888 | 2.223434 | Down-Regulated |
| CTNNBIP1 | -0.75198 | 10.8575 | -4.18021 | 7.38E-05 | 0.009971 | 1.419388 | Down-Regulated |
| C1orf186 | -1.63195 | 4.460781 | -3.78424 | 0.000296 | 0.031506 | 0.141757 | Down-Regulated |
| MAGIX | -0.73918 | 5.330037 | -3.75132 | 0.000331 | 0.034696 | 0.039388 | Down-Regulated |
| GATSL3 | -0.78269 | 7.679304 | -3.696 | 0.000399 | 0.040153 | -0.13126 | Down-Regulated |
| GLTPD1 | -0.56822 | 9.832281 | -3.68429 | 0.000415 | 0.041182 | -0.16717 | Down-Regulated |
| LDLRAP1 | -0.50561 | 9.920141 | -3.67887 | 0.000423 | 0.041474 | -0.18376 | Down-Regulated |

**Supplementary Table 2** Differences in IC50 levels of 138 chemotherapeutic agents.

| **DrugName** | **p-value** |
| --- | --- |
| BX.795 | 6.71E-07 |
| Pazopanib | 2.64E-06 |
| PLX4720 | 4.36E-06 |
| Imatinib | 5.58E-06 |
| NU.7441 | 6.15E-06 |
| GSK269962A | 9.91E-06 |
| CEP.701 | 5.87E-05 |
| AG.014699 | 0.000161 |
| XMD8.85 | 0.00022 |
| WO2009093972 | 0.000256 |
| KIN001.135 | 0.000308 |
| WH.4.023 | 0.000357 |
| SB.216763 | 0.000512 |
| Z.LLNle.CHO | 0.000512 |
| Midostaurin | 0.000569 |
| Bexarotene | 0.000953 |
| AP.24534 | 0.001203 |
| Embelin | 0.001243 |
| CHIR.99021 | 0.002345 |
| Dasatinib | 0.003677 |
| AZ628 | 0.004766 |
| GDC0941 | 0.004766 |
| PF.02341066 | 0.005335 |
| VX.702 | 0.005963 |
| BMS.754807 | 0.00613 |
| A.770041 | 0.008472 |
| Axitinib | 0.01246 |
| AZD7762 | 0.016322 |
| Cyclopamine | 0.018825 |
| AZD6482 | 0.019273 |
| PHA.665752 | 0.021644 |
| BMS.708163 | 0.022147 |
| AZD.2281 | 0.023181 |
| Parthenolide | 0.025944 |
| OSI.906 | 0.026528 |
| NVP.BEZ235 | 0.045931 |
| BMS.509744 | 0.049728 |
| NVP.TAE684 | 0.05377 |
| X17.AAG | 0.054819 |
| AZD.0530 | 0.060308 |
| CI.1040 | 0.0638 |
| DMOG | 0.069963 |
| GDC.0449 | 0.077944 |
| ABT.263 | 0.091169 |
| PD.173074 | 0.104245 |
| LFM.A13 | 0.111264 |
| CGP.60474 | 0.114897 |
| Sunitinib | 0.144806 |
| Elesclomol | 0.146969 |
| ZM.447439 | 0.149153 |
| FTI.277 | 0.174591 |
| TW.37 | 0.181976 |
| Bicalutamide | 0.187004 |
| MK.2206 | 0.18955 |
| RDEA119 | 0.18955 |
| AUY922 | 0.202593 |
| Bryostatin.1 | 0.241822 |
| Cytarabine | 0.250729 |
| JNJ.26854165 | 0.259804 |
| AMG.706 | 0.262866 |
| BMS.536924 | 0.300974 |
| AS601245 | 0.304257 |
| AZD8055 | 0.304257 |
| CCT007093 | 0.32767 |
| PAC.1 | 0.355268 |
| CMK | 0.38723 |
| Docetaxel | 0.394446 |
| BAY.61.3606 | 0.398067 |
| BI.D1870 | 0.401697 |
| PF.562271 | 0.401697 |
| Temsirolimus | 0.401697 |
| Vorinostat | 0.445828 |
| KU.55933 | 0.449543 |
| Bleomycin | 0.460715 |
| X681640 | 0.46818 |
| IPA.3 | 0.486886 |
| Nilotinib | 0.509368 |
| GNF.2 | 0.524344 |
| Nutlin.3a | 0.524344 |
| RO.3306 | 0.56529 |
| Pyrimethamine | 0.587363 |
| MS.275 | 0.658636 |
| JNK.Inhibitor.VIII | 0.66551 |
| WZ.1.84 | 0.679093 |
| Cisplatin | 0.689128 |
| QS11 | 0.702294 |
| Shikonin | 0.702294 |
| SB590885 | 0.72784 |
| Obatoclax.Mesylate | 0.737134 |
| Camptothecin | 0.740196 |
| MG.132 | 0.740196 |
| NSC.87877 | 0.775469 |
| CGP.082996 | 0.786601 |
| BI.2536 | 0.797407 |
| AZD6244 | 0.800057 |
| PD.0325901 | 0.800057 |
| Bortezomib | 0.802687 |
| Doxorubicin | 0.815521 |
| FH535 | 0.844165 |
| JNK.9L | 0.869741 |
| Gemcitabine | 0.871735 |
| CCT018159 | 0.873707 |
| Vinblastine | 0.877589 |
| Rapamycin | 0.910377 |
| GW.441756 | 0.911903 |
| Thapsigargin | 0.911903 |
| EHT.1864 | 0.928752 |
| Epothilone.B | 0.933787 |
| AICAR | 0.938545 |
| PF.4708671 | 0.938545 |
| PD.0332991 | 0.947264 |
| GW843682X | 0.956764 |
| GSK.650394 | 0.957633 |
| Lenalidomide | 0.969737 |
| SL.0101.1 | 0.972272 |
| Paclitaxel | 0.975745 |
| VX.680 | 0.976819 |
| ATRA | 0.978356 |
| Bosutinib | 0.981175 |
| Etoposide | 0.983282 |
| ABT.888 | 0.984447 |
| JW.7.52.1 | 0.984447 |
| Lapatinib | 0.98754 |
| AKT.inhibitor.VIII | 0.98815 |
| S.Trityl.L.cysteine | 0.989295 |
| Salubrinal | 0.990591 |
| A.443654 | 0.991749 |
| Sorafenib | 0.991965 |
| Tipifarnib | 0.992974 |
| Roscovitine | 0.993525 |
| Vinorelbine | 0.994037 |
| Mitomycin.C | 0.995869 |
| BIRB.0796 | 0.997991 |
| Methotrexate | 0.997991 |
| Erlotinib | 0.998338 |
| BIBW2992 | 0.999556 |
| Gefitinib | 0.999984 |
| Metformin | 0.999999 |

**Supplementary Figure 1**





**Supplementary Figure 2**


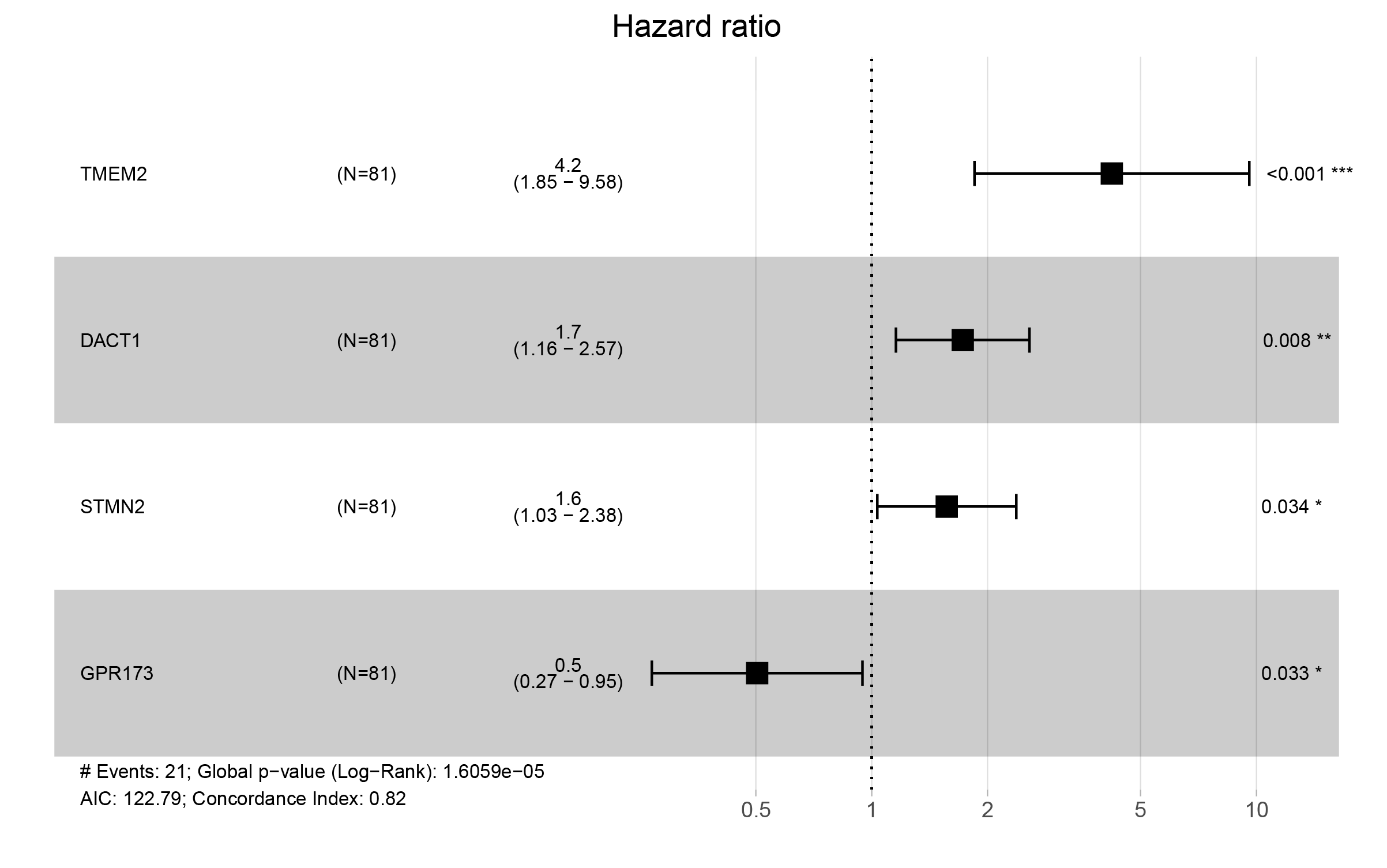


**Supplementary Figure 3**


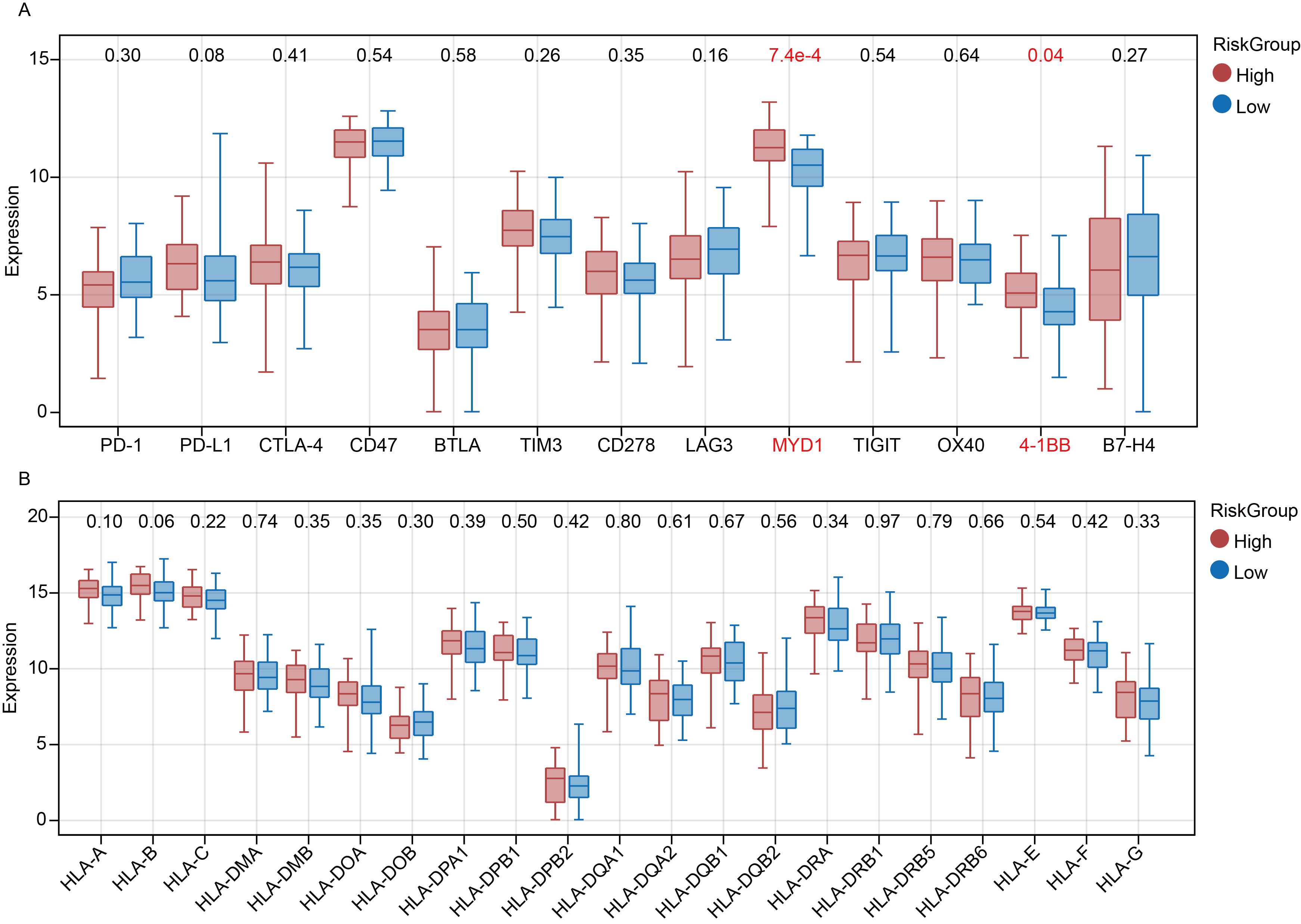

Supplement: Supplementary file 1 [file mmc1.doc]
